# GRF–GIF chimeric proteins enhance in vitro regeneration and *Agrobacterium*-mediated transformation efficiencies of lettuce (*Lactuca* spp.)

**DOI:** 10.1007/s00299-023-02980-4

**Published:** 2023-01-25

**Authors:** Tawni Bull, Juan Debernardi, Megan Reeves, Theresa Hill, Lien Bertier, Allen Van Deynze, Richard Michelmore

**Affiliations:** 1 Graduate Group in Horticulture and Agronomy, University of California, Davis, Davis, CA USA; 2 The Genome Center, University of California, Davis, Davis, CA USA; 3 Plant Transformation Facility, University of California, Davis, CA USA; 4 The Seed Biotechnology Center, University of California, Davis, Davis, CA USA; 5 Department of Plant Sciences, University of California, Davis, Davis, CA USA; 6 Present Address: Department of Biology, Colorado State University, Fort Collins, CO USA; 7 Present Address: Ohalo Genetics, Inc., Aptos, CA USA

**Keywords:** Regeneration, GRF, GROWTH-REGULATING FACTOR, GIF, Lettuce

## Abstract

**Key message:**

**GRF–GIF chimeric proteins from multiple source species enhance in vitro regeneration in both wild and cultivated lettuce. In addition, they enhance regeneration in multiple types of lettuce including butterheads, romaines, and crispheads**.

**Abstract:**

The ability of plants to regenerate in vitro has been exploited for use in tissue culture systems for plant propagation, plant transformation, and genome editing. The success of in vitro regeneration is often genotype dependent and continues to be a bottleneck for *Agrobacterium*-mediated transformation and its deployment for improvement of some crop species. Manipulation of transcription factors that play key roles in plant development such as BABY BOOM, WUSCHEL, and GROWTH-REGULATING FACTORs (GRFs) has improved regeneration and transformation efficiencies in several plant species. Here, we compare the efficacy of *GRF–GIF* gene fusions from multiple species to boost regeneration efficiency and shooting frequency in four genotypes of wild and cultivated lettuce (*Lactuca *spp. L.). In addition, we show that GRF–GIFs with mutated miRNA 396 binding sites increase regeneration efficiency and shooting frequency when compared to controls. We also present a co-transformation strategy for increased transformation efficiency and recovery of transgenic plants harboring a gene of interest. This strategy will enhance the recovery of transgenic plants of other lettuce genotypes and likely other crops in the Compositae family.

**Supplementary Information:**

The online version contains supplementary material available at 10.1007/s00299-023-02980-4.

## Introduction

Plants have an incredible capacity to regenerate whole organs from differentiated cells through dedifferentiation and reprogramming of cells. This capability has been exploited for use in tissue culture systems for plant propagation, plant transformation, and genome editing (Altpeter et al. 2016). The success of in vitro regeneration is often genotype dependent and continues to be a bottleneck for *Agrobacterium*-mediated transformation and its implementation for improvement of some crop species. Multiple species are capable of regenerating (e.g., lettuce, tomato, *Arabidopsis*) but many high value crops, such as cotton, sunflower, and pepper, are still recalcitrant to regeneration (Gammoudi et al. 2018; Wu et al. 2004). Previously, research has been conducted to identify methods to improve somatic embryogenesis and de novo organogenesis for the development of transgenic lines (Debernardi et al. 2020; Elhiti et al. 2021; Jones et al. 2019; Kong et al. 2020; Stasolla and Yeung 2003; Zheng et al. 2013). For example, incorporation of several genes encoding developmental regulators such as *LEAFY COTYLEDON1* (*LEC1*), *LEC2*, *WUSCHEL* (*WUS*), and *BABY BOOM* (*BBM*) increases somatic embryogenesis by promoting vegetative to embryonic cell transition (Jones et al. 2019). Although the use of these genes increased regeneration rates, unregulated ectopic expression also induced pleiotropic phenotypes affecting cotyledon, hypocotyl, shoot development, and fertility (Boutilier et al. 2002). Pathways involved in in vitro regeneration include those for plant development; therefore, the use of other developmental genes regulating cell proliferation and organ development should be explored for increasing regeneration of recalcitrant species.

GROWTH-REGULATING FACTOR (GRF) transcription factors are involved in regulating multiple stages of plant development including leaf, stem, root, seed, and flower development. GRFs tend to be associated with tissues of prolific cell division during development (reviewed in Omidbakhshfard et al. 2015; Rodriguez et al. 2016). The first GRF, *OsGRF1*, was identified in rice and was observed to play a role in gibberellic acid induced stem elongation (van der Knaap et al. 2000). Now, multiple GRFs have been identified and studied in both monocotyledonous and dicotyledonous species including rice, barley, wheat, maize, *Arabidopsis, Brassica* spp., tomato, potato, and lettuce (Huang et al. 2021; Khatun et al. 2017; Li et al. 2016; Ma et al. 2017; Rosenquist et al. 2001; Wang et al. 2014; B. Zhang et al. 2021; D. F. Zhang et al. 2008). Individual species tend to have multiple GRFs each with different developmental functions and most with two conserved domains. One domain, the QLQ domain, likely functions in protein–protein interactions, and the second domain, the WRC domain, may function in nuclear targeting and DNA binding (Omidbakhshfard et al. 2015; van der Knaap et al. 2000). GRFs have a transcriptional co-factor, GRF-INTERACTING FACTOR (GIF), which interacts with the QLQ domain and enhances the function of GRFs (Debernardi et al. 2014). GRFs are also post-transcriptionally regulated by microRNA 396 (miR396), which recognizes and binds to the nucleotides of the WRC domain (Rodriguez et al. 2010). These three components together make up what is known as the miR396-GRF/GIF module in plant development (Omidbakhshfard et al. 2015).

Transformation using a *GRF* only or a *GRF–GIF* chimeric transgene increased regeneration efficiency in both monocotyledonous and dicotyledonous species (Debernardi et al. 2020; Kong et al. 2020). The ectopic expression of *AtGRF5* increased transgenic callus formation in canola and shoot organogenesis in multiple varieties of sugar beet, soybean, and sunflower (Kong et al. 2020). In addition, overexpression of *GRF* orthologs in each species resulted in boosted shoot organogenesis and transformation efficiency. The overexpression of maize *AtGRF* orthologs were also shown to increase formation of embryogenic calli leading to higher levels of regeneration by somatic embryogenesis in maize (Kong et al. 2020). Furthermore, the use of GRFs in conjunction with GIFs also enhanced regeneration frequencies and rates in wheat, triticale, rice, and citrus (Debernardi et al. 2020). Transformations using species specific homologs of the *GRF4*–*GIF1* chimeric transgene resulted in better regeneration when compared to transformations of *GRF4* only, *GIF1* only, and a co-transformation of *GRF4* and *GIF1*. Transgenic wheat plants were also recovered in the absence of antibiotic-based selectable markers and hormones. In addition, citrus and grape *GRF4*–*GIF1* orthologs were tested in citrus and resulted in an approximately fivefold increase in regeneration (Debernardi et al. 2020). A microRNA-resistant grape *GRF–GIF* (r*GRF–GIF)* gene fusion containing synonymous mutations in the miR396 binding site to prevent post-transcriptional degradation of the GRF mRNA was also transformed into citrus, which resulted in the highest regeneration efficiency, although developmental defects were observed. Lastly, adding a *GRF4–GIF1* fusion into an editing construct resulted in a large number of independent editing events in wheat (Debernardi et al. 2020). Both studies observed an increase in regeneration of recalcitrant or low-transforming cultivars and resulted in developmentally normal plants.

In this paper, we tested *GRF–GIF* chimeric transgenes from different source species and characterized their ability to increase regeneration and transformation efficiency in multiple lettuce (*Lactuca* spp.) genotypes. The aim of this study was to answer four questions: (1) *GRF–GIF* chimeric transgenes from which species most enhances regeneration and shooting frequency in lettuce? (2) Does mutation of the miR396 binding site of the *GRF* fragment alter regeneration and transformation efficiency when compared to the wild-type *GRF* coding sequence? (3) Is enhancement of regeneration efficiency and shooting frequency using *GRF–GIF* gene fusions genotype-independent in lettuce? (4) What plant selection and co-transformation strategy is most efficient when using *GRF–GIFs* for enhancing transformation rates with a gene of interest? The results of this study provide further evidence that the introduction of *GRF–GIF* chimeric transgenes can increase regeneration and transformation efficiency, even across plant families. In addition, a new co-transformation strategy was demonstrated for increasing the efficiency for introducing genes of interest into lettuce.

## Materials and methods

### Vectors and vector construction

The miRNA-resistant chimeric *GRF4–GIF1* coding sequences from grape and the wild-type fusion from citrus used for this study were as described previously (Debernardi et al. 2020). To identify the homologous *GRF4* and *GIF1* genes from tomato (Solanaceae), phylogenetic trees were generated using GRF and GIF protein sequences from wheat, rice, *Arabidopsis*, *Citrus*, grape (*Vitis vinifera*), and tomato (*Solanum lycopersicum*); the QLQ and WRC domains were used for GRF protein sequences and the SNH domain was used for GIF protein sequences. The evolutionary history was inferred by using the maximum likelihood method (Supplemental Fig. 1). A BLAST search with wheat and grape *GRF* and *GIF* genes was used to identify *GRF* and *GIF* genes in pepper (*Capsicum annuum*); pepper GRF protein sequences were aligned with GRF protein sequences from citrus, grape, *Arabidopsis*, and *Medicago truncatula*. Pepper GIF protein sequences were aligned with GIF protein sequences from several species. Both alignments were generated using T-Coffee (https://tcoffee.crg.eu/) with the M-Coffee aligner. Phylogenetic trees were generated using MEGA5 (Supplemental Fig. 2). GRF and GIF protein sequences most closely related to the grape *GRF4* and *GIF1* sequences were used to synthesize the *GRF–GIF* fusion constructs. In tomato, we selected two closely related GRFs (Solyc08g075950 and Solyc12g096070), which were called GRF4#8 and GRF4#12 based on the chromosome they are located. These chimeric sequences were developed by fusing the *GRF* and *GIF* coding sequences with a four-alanine linker and synthesized by Genewiz (https://www.genewiz.com/en).

The miR396-resistant versions of tomato *GRF–GIF* (*rGRF–GIF*) were generated by overlapping PCR. For each GRF, two PCR reactions were performed with primers Fw-Gw/rGRF-Rev and rGRF-Fw/Rev-GW (Supplemental Table S3) using tomato *GRF4#8–GIF1* and *GRF4#12–GIF1* pDONR^™^/Zeo clones as templates. The pair of primers rGRF#8-Fw/rGRF#8-Rev and rGRF#12-Fw/rGRF#12-Rev overlap by 17 nucleotides and introduce the same four silent mutations in the miR396 target site as shown before for grape *rGRF4–GIF1* vector (Debernardi et al. 2020). The PCR fragments for each GRF were used as template in a second PCR with the primers Fw-Gw/Rev-Gw (Supplemental Table S3), which was then cloned in pDONR^™^/Zeo by a Gateway BP reaction.

All *GRF–GIF* fusions constructs for transformation were generated using Gateway^™^ cloning. The *GRF–GIF* fusions were first cloned into either pDONR^™^/Zeo or pDONR221. The pepper *GRF4–GIF1* chimeric coding sequence was then subcloned into pEarlyGate100 (pTH1903) using the L/R Gateway^™^ reaction. The wild-type tomato *GRF4#8–GIF4* was subcloned in pEG100 (pJD761) and pGWB14 (JD746). Resistant tomato r*GRF4#8–GIF1* (pJD747) and r*GRF4#12–GIF1* (pJD749) were subcloned into pGWB14. Empty vectors of pGWB14 (pJD641) (Debernardi et al. 2020) and pEG100 (pTB005) were used as controls for each transformation.

The promoter and terminator from the *L. sativa polyubiquitin 4* gene (LOC111919935) were cloned from cv. Salinas. The promoter region was cloned in three parts to mutate existing type IIS restriction enzyme recognition sites in order to make it Golden Gate cloning compatible. The promoter and terminators were cloned into pL1M modules using Golden Gate cloning. In a second cloning step, the pL2B binary vector was assembled from the pL1M modules (pLsUBI, dsRED and tLsUBI) to create pL2B-Kan–pLsUBI–dsRED–tLsUBI, the final binary vector used as a gene of interest reporter for *Agrobacterium* co-transformation experiments A detailed description of all constructs is provided in Table 1.

**Table 1:** Description of all constructs used in this study

Construct name	Vector backbone	Selectable marker	Origin species/GRF–GIF	Wild type/resistant	Promoter/terminator
pTB005	pEG100	BASTA (*bar*)	Empty vector	–	CaMV p35S/ tOCS
JD761	pEG100	BASTA (*bar*)	Tomato *GRF4#8–GIF1*	Wild type	CaMV p35S/tOCS
pEG100-JD638	pEG100	BASTA(*bar*)	Grape *GRF4–GIF1*	Resistant	CaMV p35S/tOCS
pEG100-JD689	pEG100	BASTA(*bar*)	Citrus *GRF–GIF*	Wild type	CaMV p35S/tOCS
pTH1903	pEG100	BASTA(*bar*)	Pepper *GRF4–GIF1*	Wild type	CaMV p35S/tOCS
JD641	pGWB14	Kanamycin (*nptII*)	Empty vector	–	CaMV p35S/tNOS
JD746	pGWB14	Kanamycin (*nptII*)	Tomato *GRF4#8–GIF1*	Wild type	CaMV p35S/tNOS
JD747	pGWB14	Kanamycin (*nptII*)	Tomato *GRF4#8–GIF1*	Resistant	CaMV p35S/tNOS
JD749	pGWB14	Kanamycin (*nptII*)	Tomato *GRF4#12–GIF1*	Resistant	CaMV p35S/tNOS
*pLsUBI–dsRED–tLsUBI*	pL2B	Kanamycin (*nptII*)	–	–	pLsUBI/tLsUBI

### Preparation of bacterial cultures

All plasmids were transformed into *Agrobacterium tumefaciens* strain LBA4404. Initial bacterial cultures were prepared by inoculating 20 mL of MGL medium (5 g/L tryptone, 2.5 g/L yeast extract, 5 g/L NaCl, 5 g/L mannitol, 0.1 g/L MgSO_4_, 0.25 g/L K_2_HPO_4_, 1.2 g/L glutamic acid, 15 g/L sucrose; pH 7.2) supplemented with rifampicin (50 mg/L) and kanamycin (50 mg/L) with one colony of *Agrobacterium tumefaciens* strain LBA4404 harboring binary plasmids required for each experiment. Cultures were incubated overnight in an orbital shaker at 28 °C at 200 rpm. The following day, subcultures were prepared by inoculating 17 mL of TY medium (5 g/L tryptone, 3 g/L yeast extract; pH 7.2) supplemented with rifampicin (50 mg/L), kanamycin (50 mg/L), and acetosyringone (40 mg/L) with three mL of the previous overnight culture. Cultures were incubated overnight in an orbital shaker at 28 °C at 200 rpm. The following morning, cultures were diluted to an OD_600_ between 0.1 and 0.2. For co-transformation, diluted bacterial cultures were mixed at a 1:1 ratio, and the OD_600_ was remeasured to ensure correct bacterial density. Acetosyringone was added to the final diluted cultures prior to transformation at a final concentration of 200 µM.

### Preparation of explants and transformations

Seeds were surface sterilized with 20% Clorox for 20 min with constant agitation at 250 rpm. Sterile seeds were rinsed three times with 100 mL of sterile distilled water and sown on 1/2 × Hoagland’s medium (0.815 g/L Hoagland modified basal salt mixture [PhytoTech Labs Product ID# H353], 8 g/L PhytoAgar^™^ [PlantMedia SKU# 40100072-1], pH 5.6–5.8). Seeds were incubated for 4 days in a 24 °C growth room under a 12/12 h light/dark cycle with Honeywell LED lights (Model #SH450505Q2004) providing approximately 8700 lx. After 4 days, explants were prepared by cutting off apical and basal tips of cotyledons while submerged in 20 mL of *A. tumefaciens* suspension culture. Cotyledon explants were immediately transferred to SH co-cultivation medium 3.2 g/L Schenk and Hilderbrandt (SH) Basal Salt Mixture [PhytoTech Labs Product ID# S816], 30 g/L sucrose, 2 mL/L 500 × Murashige and Skoog (MS) Vitamins [PhytoTech Labs Product ID# M533], 8 g/L PhytoAgar, pH 7.2) supplemented with acetosyringone (200 µM), 0.1 mg/L of 6-benzylaminopurine (6-BAP), and 0.1 mg/L of 1-naphthaleneacetic acid (1-NAA) and incubated in the dark for 3 days at 24 °C. A total of 34 independent transformations were performed with approximately 80 cotyledon explants each (Supplemental Table 1). Each independent transformation was split into five replications of approximately 16 explants per plate. Individual transformations and experiments are described in more detail below.

### Comparison of regeneration stimulated by GRF–GIF fusions from four plant species

A readily regenerating genotype, *L sativa* cv. Cobham Green, and an inconsistently regenerating genotype, *L. serriola* accession Armenian 999, were used for transformation. These genotypes were transformed using the wild-type tomato (JD761), citrus (pEG100-JD689), pepper (pTH1903), miRNA-resistant grape (pEG100-JD638) *GRF–GIF* fusions, and an empty vector control (pTB005). After incubation on co-cultivation, explants were transferred to SH Induction (SHI) medium (3.2 g/L SH basal salt mixture, 30 g/L sucrose, 2 mL/L 500 × MS vitamins, 8 g/L PhytoAgar^™^; pH 5.6–5.8; after autoclaving—0.10 mg/L 6-BAP, 0.10 mg/L 1-NAA, 150 mg/L timentin, and 400 mg/L carbenicillin were added) supplemented with 10 mg/L Glufosinate-ammonium (BASTA) and incubated in a HiPoint growth chamber (model FH-1200 LED Z4) at 26 °C under a 12/12 h light/dark cycle for two weeks. After 14 days in culture, explants were transferred to fresh SHI. After 24 and 35 days in culture for Cobham Green and Armenian 999, respectively, explants showing regeneration were transferred to SH elongation (SHE) medium (3.2 g/L SH Basal Salt mixture, 30 g/L sucrose, 2 mL/L 500 × MS vitamins, 8 g/L PhytoAgar^M^, pH 5.6–5.8; after autoclaving—0.01 mg/L 6-BAP, 0.05 mg/L 1-NAA, and 150 mg/L Timentin were added) supplemented with 10 mg/L BASTA and returned to the growth chamber. The final shooting frequency and regeneration efficiency was calculated after 35 and 45 days in culture for Cobham Green and Armenian 999, respectively.

### Analysis of regeneration stimulated by wild-type or miRNA-resistant GRF–GIFs

To test the efficacy of regeneration using wild-type *GRF–GIF* and r*GRF–GIF* fusions, Cobham Green and Armenian 999 cultivars were used for transformation. These two genotypes were transformed with the wild-type tomato *GRF4#8–GIF1*(JD746), the tomato r*GRF4#8–GIF1* (JD747), the tomato r*GRF4#12–GIF1* (JD749), and an empty vector control (JD641). After incubation on co-cultivation medium, explants were transferred to SHI medium supplemented with 100 mg/L kanamycin and incubated in a HiPoint growth chamber (model FH-1200 LED Z4) at 26 °C under a 12/12 h light/dark cycle for 2 weeks. After 14 days on SHI, explants were transferred to fresh SHI. After 21 days in culture for Cobham Green and 27 days in culture for Armenian 999, all explants exhibiting regeneration were transferred onto SHE medium and returned to the growth chamber. After 35 and 45 days in culture for Cobham Green and Armenian 999, respectively, the shooting frequencies and regeneration efficiencies were calculated.

### The effect of lettuce genotype on regeneration using rGRF–GIF

The lettuce cultivars, Cobham Green (butterhead), Salinas (crisphead), Valmaine (romaine), and the *L. serriola* accession, Armenian 999, were used for transformation. Each genotype was transformed with the best performing GRF–GIF fusion, grape r*GRF4–GIF1*, and an empty vector control (pTB005). After co-cultivation, cultures were transferred to SHI supplemented with BASTA (10 mg/L) and incubated in a 24 °C growth room with Honeywell LED lights (Model #SH450505Q2004) providing approximately 8700 lx and under a 12/12 h light/dark cycle for 20 days. After 20 days on induction medium, explants were transferred to new SHI supplemented with BASTA (10 mg/L) and returned to the growth room. After 40 days on induction medium, all explants exhibiting regeneration in Cobham Green, Valmaine, Salinas, and Armenian 999 cultures were transferred to SHE, followed by collection of shooting frequency and regeneration efficiency for each replication.

### Co-transformation strategies to generate transgenics with genes of interest using rGRF–GIF

The grape r*GRF4–GIF1* (pEG100-JD638) was used to test for increased regeneration of transgenics with a gene of interest using *dsRED* expressed from the *L. sativa* ubiquitin promoter and terminator (*pLsUBI:dsRED:tLsUBI*) and carried in a separate LBA4404 strain of *A. tumefaciens*. *A. tumefaciens* cultures were prepared for transformation as described above. Co-transformation cultures were prepared by mixing diluted cultures of pEG100-JD638 and *pLsUBI:dsRED:tLsUBI* in a 1:1 ratio to give a final OD_600_ ranging from 0.139 to 0.145. Explants were prepared and co-transformed using two mixtures: (1) pEG100-JD638 + *pLsUBI:dsRED:tLsUBI* and (2) pTB005 + *pLsUBI:dsRED:tLsUBI* (Table 2).

**Table 2:** Co-transformation treatments and selection strategies

Co-transformation	Constructs	Selection
coTF BASTA	*pLsUBI–dsRED–tLSUBI* + pEG100-JD638	BASTA
coTF Kan	*pLsUBI–dsRED–tLSUBI* + pEG100-JD638	Kanamycin
coTF Kan + BASTA	*pLsUBI–dsRED–tLSUBI* + pEG100-JD638	BASTA + Kanamycin
coTF control	*pLsUBI–dsRED–tLSUBI* + pTB005	Kanamycin

Four co-transformations of *L. sativa* cv. Cobham Green and *L. serriola* accession Armenian 999 each were performed as described above. For each genotype, three transformations were performed using mixture 1 and one transformation using mixture 2. After co-cultivation, explants were transferred to SHI medium consisting of different antibiotic-based selection regimes. The three co-transformations using mixture 1 were transferred to SHI medium supplemented with either (1) 100 mg/L kanamycin (further referred to as coTF Kan) to select for all cells successfully transformed with the gene of interest (*dsRED*), (2) 10 mg/L BASTA (further referred to as coTF BASTA) to select for all cells successfully transformed with the grape r*GRF–GIF*, or (3) 100 mg/L kanamycin and 10 mg/L BASTA (further referred to as coTF Kan + BASTA) to select for events only transformed with both T-DNAs. The single transformation using mixture 2 was selected on SHI supplemented with 100 mg/L kanamycin (further referred to as coTF Control) to act as a control for transformation rate in the absence of GRF–GIF.

Maintenance of tissue cultures was performed as described for previous transformation experiments. After incubation on co-cultivation, explants were transferred to SHI supplemented with the different antibiotics and incubated in a HiPoint growth chamber (model FH-1200 LED Z4) at 26 °C under a 12/12 h light/dark cycle for 2 weeks. After 14 days in culture, explants from all transformations were transferred to fresh SHI supplemented with respective antibiotics and returned to the growth chamber. After 20 and 35 days on SHI medium for Cobham Green and Armenian 999, respectively, explants showing regeneration were transferred to SHE medium supplemented with respective antibiotics and the cultures were returned to the growth chamber. After 30 and 45 days in culture for Cobham Green and Armenian 999, respectively, all regenerated shoots were transferred to rooting media supplemented with respective antibiotics, the shooting frequency and regeneration efficiency data were collected during the transfer.

### DNA extraction and screening of transgenics

DNA was extracted from leaf tissue (~ 10 to 30 mg) of the regenerated plants using the QIAGEN DNeasy^®^ Plant Mini Kit (Cat. No. 69104). DNA was extracted from leaf tissue of regenerated shoots from coTF Kan to screen for the presence of the grape r*GRF4–GIF1*, *dsRED*, *bar* (BASTA), and *nptII* (Kanamycin) transgenes. PCR amplification of each fragment was performed using Promega GoTaq^®^ Green Master Mix. Primer sequences and PCR conditions for each reaction are described in Supplemental Table 2. In addition, this data was used to calculate co-transformation efficiency for all the Armenian 999 and Cobham Green genotypes. Furthermore, for the co-transformation experiments, the expression of *dsRED* was observed using the Leica MZ16NF dissecting microscope and a Chroma^Ⓡ^ dsRED filter (Product # 49004; filter ET605/70 m). Frequencies of *dsRED* expression were used to calculate transformation efficiencies of coTF Kan, coTF Kan + BASTA, and coTF control, and the co-transformation efficiency of coTF BASTA.

### Data analysis

For every replication of each transformation, regeneration efficiencies were determined by dividing the total number of explants with at least one shoot by the number of co-cultivated explants and multiplied by 100. Transformation and co-transformation efficiencies were determined by dividing the total number of explants with at least one shoot containing one or both (co-transformation) transgenes by the total number of explants inoculated and multiplied by 100. Organized growth and leaf emergence efficiencies were calculated by dividing the total number of explants exhibiting each trait by the number of inoculated explants and multiplied by 100. Shooting frequencies were calculated by dividing the total number of shoots or transgenic shoots by the total number of inoculated explants and multiplied by 100. Analysis of variance (ANOVA, *α* = 0.05) was used to observe significant differences between regeneration traits (organized growth, leaf emergence, and shoot frequency) and regeneration and transformation efficiencies of each transformation experiment. A Tukey’s honest significant difference (Tukey HSD, *α* = 0.05) test was used to calculate all pairwise comparisons for each batch of transformations. For the genotype independence experiments, a Welch’s *t* test (*α* = 0.05) was used to compare means between the empty vector control and the grape *GRF4–GIF1* for each genotype. Data analysis and visualization was performed using Microsoft Excel version 2202 and RStudio version 2021.09.1 + 372.

## Results

### Comparison of regeneration stimulated by GRF–GIF fusions from four plant species

*GRF4–GIF1* chimeric transgenes from four dicotyledonous species [tomato (*GRF4#8–GIF1*), pepper (*GRF4–GIF1*), citrus (*GRF4–GIF1*), and grape (r*GRF4–GIF1*)] were tested for their effect on regeneration efficiency and shooting frequency in two genotypes of lettuce: Cobham Green and *L. serriola* accession Armenian 999, which regenerate readily and inconsistently, respectively (Table 3). Final shooting frequency and regeneration efficiency data were collected for Cobham Green and Armenian 999 after 35 and 45 days in culture, respectively, and after being transferred to elongation medium.

**Table 3:** Means of final shooting frequencies (shoots per explant) and regeneration efficiencies (%) of each treatment from the GRF–GIF experiments in Armenian 999 and Cobham Green

Variety	Construct	Mean shooting frequency	ANOVA	TukeyHSD	Mean RE	ANOVA	TukeyHSD
Armenian 999	pTB005	0.37	0.0002***	a	11.42	0.00008***	a
	Tomato	0.42		a	15.73		ab
	Pepper	0.57		a	14.71		ab
	Citrus	0.91		a	33.73		bc
	rGrape	1.68		b	53.01		c
	JD641	0.82	0.0008***	a	46.92	0.0004***	a
	Tomato WT *GRF8–GIF4*	1.33		ab	48.75		a
	Tomato r*GRF8–GIF4*	1.94		b	69.19		b
	Tomato r*GRF12–GIF4*	1.96		b	68.33		b
Cobham Green	pTB005	0.58	6.19 × 10^–9^***	a	36.25	0.0002***	a
	Tomato	0.64		a	41.25		a
	Pepper	1.40		b	56.25		ab
	Citrus	1.51		b	55.0		ab
	rGrape	2.32		c	75.15		b
	JD641	0.80	0.0006***	a	45.00	0.005**	a
	Tomato WT *GRF8–GIF4*	1.05		ab	48.24		ab
	Tomato r*GRF8–GIF4*	1.53		c	71.92		c
	Tomato r*GRF12–GIF4*	1.46		bc	67.25		bc

Significant differences (*p* values) of treatments for each experiment (one-way ANOVA, *α* = 0.05) and pairwise comparisons between each treatment (TukeyHSD) for each experiment

* *p* ≤ 0.05, ** *p* ≤ 0.01, *** *p* ≤ 0.001

**Fig. 1:**
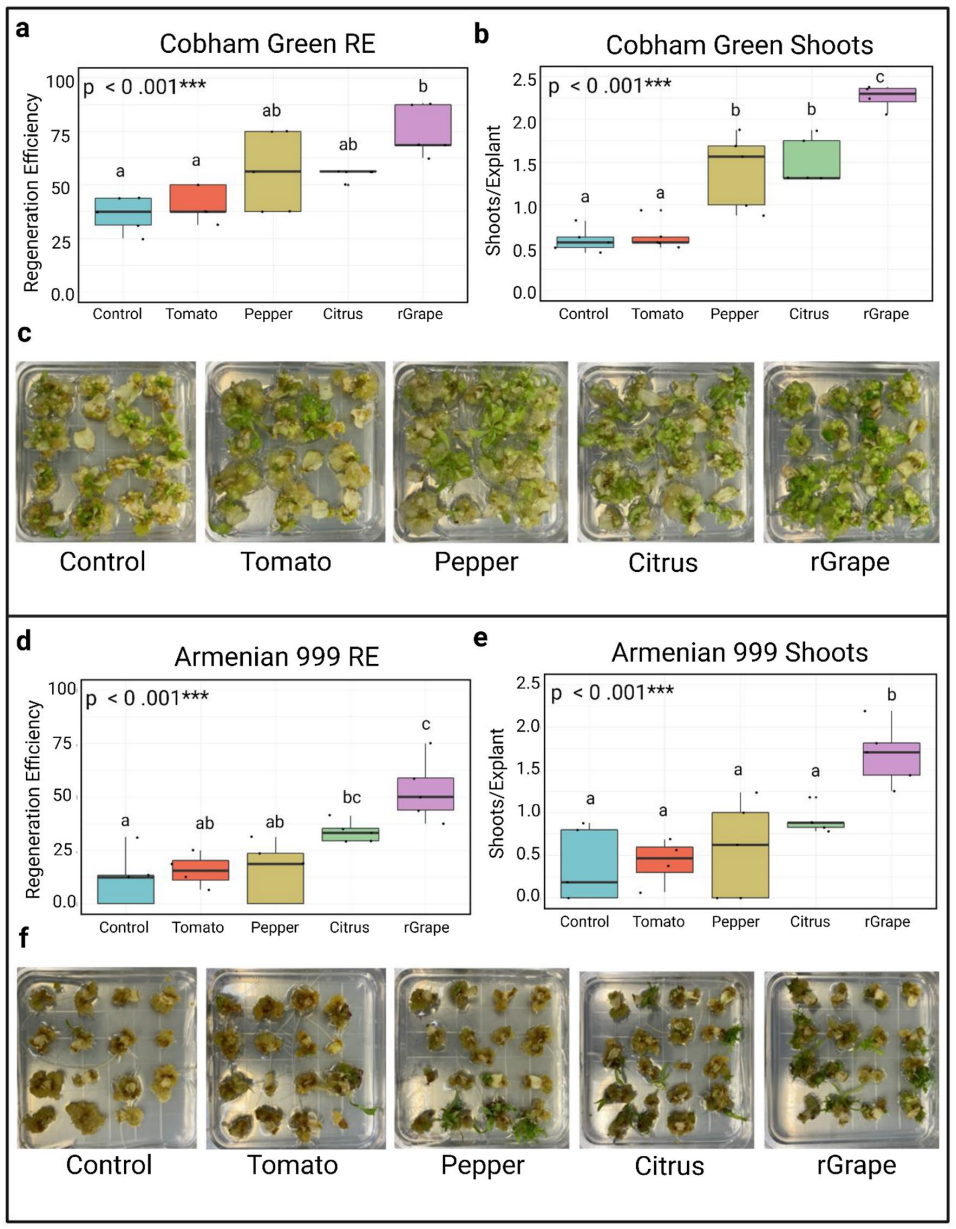
Regeneration rates of Cobham Green and Armenian 999 after transformation with *GRF–GIF* fusions from tomato, pepper, citrus, and grape. **a**, **b** Box plots representing regeneration efficiency (**a**) and shooting frequency (**b**) of Cobham Green after 35 days in culture. **c** Tissue cultures of Cobham Green after 24 days on induction medium. **d**, **e** Box plots representing regeneration efficiency (**d**) and shooting frequency (**e**) of Armenian 999 after 45 days in culture. **f** Tissue cultures of Armenian 999 after 35 days on induction medium. Letters above each boxplot represent pairwise significance differences (Tukey HSD, *α* = 0.05) and *p* values were calculated using a one-way ANOVA (*α* = 0.05) (color figure online)

In the easily transformable cultivar Cobham Green, introduction of the grape r*GRF4–GIF1* fusion resulted in approximately a 2.1-fold increase in regeneration efficiency (75.1%) and a fourfold increase in shooting frequency (2.32 shoots/explant) when compared to the control (36.3%, 0.59 shoots/explant) and the tomato GRF4#8–GIF1 (41.2%, 0.64 shoots/explant) (Fig. 1a–c; Table 3). Furthermore, the citrus (1.51 shoots/explant) and pepper (1.40 shoots/explant) *GRF–GIFs* exhibited an increase in shooting frequencies when compared to both the control and tomato *GRF4#8–GIF1*, although at lower rates than the grape *rGRF4–GIF1* (Fig. 1a–c; Table 3).

Interestingly, introduction of the grape r*GRF4–GIF1* into the more difficult cultivar to transform, Armenian 999, also resulted in significantly higher shooting frequency (1.68 shoots/explant) when compared to all other transformations, and a significant increase in regeneration efficiency (53.0%) when compared to the pepper *GRF4–GIF1* (14.7%), tomato *GRF4#8–GIF1* (15.7%), and the control (11.4%) (Fig. 1d–e; Table 3). This was approximately a 4.5- and 4.6-fold increase in shooting frequency and regeneration efficiency when compared to the empty vector control (0.37 shoots/explant, 11.4%).

Overall, for both cultivars, Cobham Green and Armenian 999, the introduction of *GRF–GIF* chimeric transgenes from other species resulted in an increase in shooting frequency and regeneration efficiency. However, the grape r*GRF4–GIF1 *that includes synonymous mutations that impair miR396 regulation resulted in the highest rates in both genotypes, suggesting that miR396 regulation could limit the activity of GRF–GIF genes in lettuce.

### Analysis of regeneration stimulated by wild-type or miRNA-resistant GRF–GIFs

Chimeric GRF–GIF fusions from tomato were used to investigate whether synonymous mutations within the miR396 binding site (r*GRF–GIF*) would enhance regeneration when compared to the wild-type GRF–GIF sequences. Cobham Green and Armenian 999 were transformed with four different constructs containing either the wild-type tomato *GRF4#8–GIF1*, the tomato r*GRF4#8–GIF4*, the tomato r*GRF4#12–GIF4*, or an empty vector control (JD641).

**Fig. 2:**
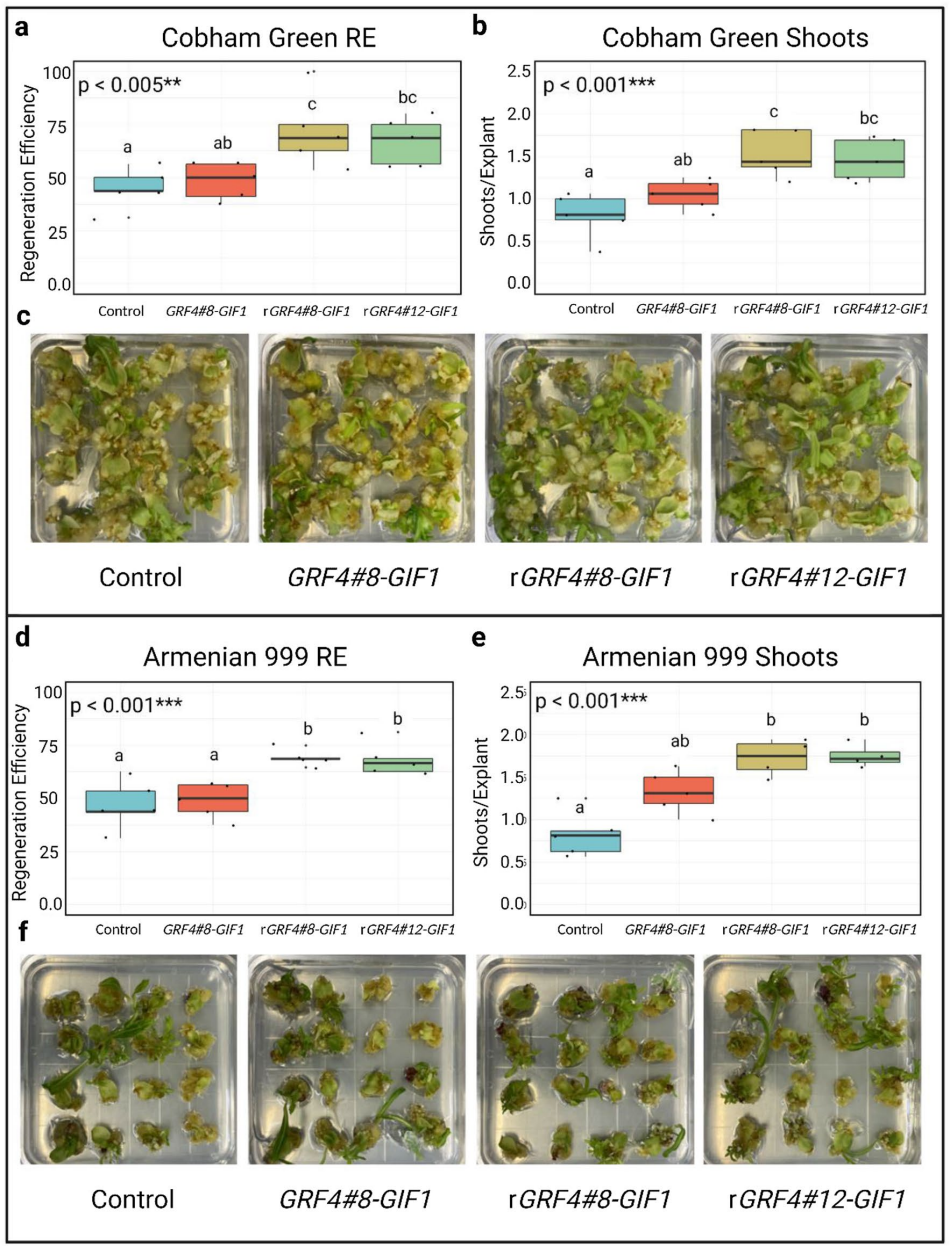
Regeneration rates of Cobham Green and Armenian 999 after introduction of the wild-type and miR396 resistant tomato *GRF–GIF* fusions. **a**, **b** Box plots representing regeneration efficiency (**a**) and shooting frequency (**b**) of Cobham Green after 35 days in culture. **c** Tissue cultures of Cobham Green after 21 days on induction medium. **d**, **e** Box plots representing regeneration efficiency (**d**) of and shooting frequency (**e**) of Armenian 999 after 45 days in culture. **f** Tissue cultures of Armenian 999 after 30 days on induction medium. Letters above each boxplot represent pairwise significance differences (TukeyHSD, *α* = 0.05) and *p* values were calculated using a one-way ANOVA (*α* = 0.05) (color figure online)

Significant differences in shooting frequency and regeneration efficiency were observed after 35 days in culture for Cobham Green and after 45 days in Armenian 999 (Fig. 2). In Cobham Green, mean regeneration efficiencies were 44.0% for both *rGRF4#8–GIF1* and *rGRF4#12–GIF1*, which is approximately 4.7% and 5.7% higher than the regeneration efficiencies observed for the wild-type *GRF4#8–GIF1* and the control, respectively (Table 3; Fig. 2a–c). In addition, the *rGRF4#8–GIF1* and *rGRF4#12–GIF1* produced approximately 1.4-fold more shoots in Cobham Green than both the wild-type *GRF4#8–GIF1* and controls. In Armenian 999, both rGRF–GIFs had approximately 20% higher regeneration efficiencies and 1.5 and 2.4-fold higher shooting frequencies than the wild-type GRF–GIF and the empty vector control, respectively (Fig. 2d, e). This is similar to the increase in shooting frequency between miR396 resistant and wild-type GRF–GIFs as seen in Cobham Green. In these experiments, no significant difference in regeneration and shooting frequencies were observed between genotypes and no significant interactions between genotype and constructs were detected (Table 4).

**Table 4:** Calculated *p* values (one-way ANOVA, *α* = 0.05) after comparing final shooting frequencies and regeneration efficiencies of constructs, genotypes, and a construct by genotype interaction

Experiment	Antibiotic selection	Factor	Shooting frequency *p* value	Regeneration efficiency *p* value
GRF–GIF stimulation from different species	BASTA	Construct	3.23 × 10^–12^***	1.42 × 10^–8^***
		Genotype	4.08 × 10^–6^***	4.44 × 10^–10^***
		Interaction	0.178	0.287
Wild type vs. rGRF–GIF	Kanamycin	Construct	5.04 × 10^–7^***	2.27 × 10^–6^***
		Genotype	0.007**	0.953
		Interaction	0.409	0.962

*P* values are shown for the first and second experiments testing the stimulation of regeneration using GRF–GIF fusions from different species and a comparison of the stimulation of wild-type and resistant GRF–GIFs with a mutated miRNA binding site

* *p* ≤ 0.05, ** *p* ≤ 0.01, *** *p* ≤ 0.001

### Effects of lettuce genotype on regeneration using rGRF–GIF

**Fig. 3:**
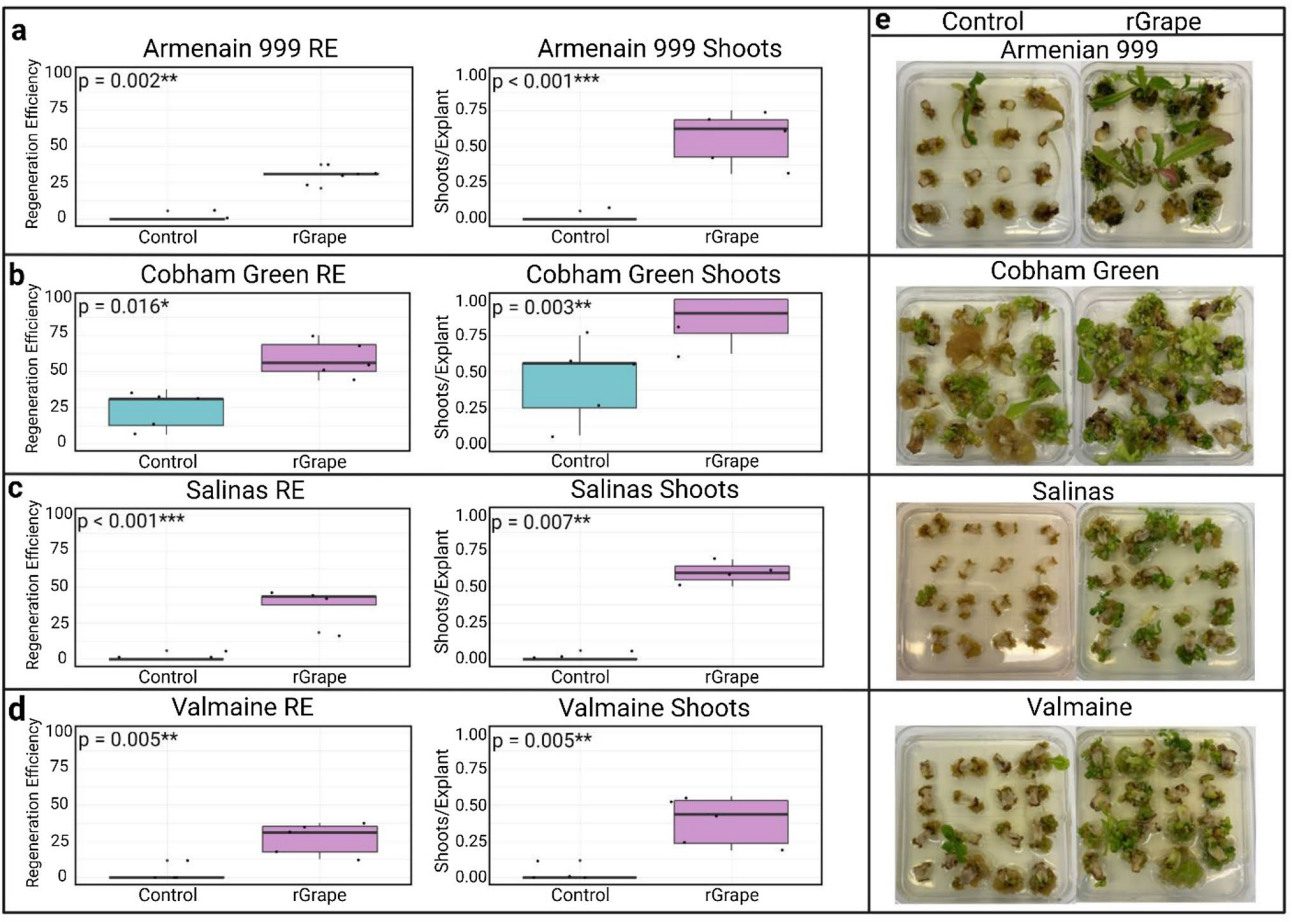
Regeneration rates of different lettuce genotypes after transformation with the grape r*GRF4–GIF1* or an empty vector control. **a**–**d** Boxplots represent regeneration efficiency (RE) and shooting frequencies for each transformation of Cobham Green (**a**), Armenian 999 (**b**), Salinas (**c**), Valmaine (**d**) after 40 days on induction medium. *p* values were calculated using a Welch’s *t* test. **e** Forty-day-old tissue cultures of each genotype after introduction of an empty vector control (left) and the grape r*GRF4–GIF1* fusion (right) (color figure online)

To test the efficacy of the GRF–GIF system in multiple types of lettuce, we transformed the highest performing *GRF–GIF* (grape r*GRF4–GIF1*) or an empty vector control (JD641) into four genotypes of lettuce: Cobham Green (a butterhead cultivar), Salinas (a crisphead cultivar), Valmaine (a romaine cultivar), and *L. serriola* Armenian 999 (a wild accession). After 40 days on induction medium, the grape *rGRF4–GIF1* significantly increased shooting frequency and regeneration efficiency of all genotypes when compared to the empty vector control (Fig. 3). Introduction of the grape r*GRF4–GIF1* into Cobham Green resulted in a 2.1- and a 2.5-fold increase in shooting frequency and regeneration efficiency when compared to the empty vector control (Table 3). In Armenian 999 cultures, transformation with the grape r*GRF4–GIF1* led to a 0.55 increase in shoots per explant and a 29.4% increase in regeneration efficiency when compared to the control. These values varied from the values observed in the first experiment (Fig. 1), which was most likely due to environmental differences (e.g., temperature, lights) between growth chambers. In Salinas, a significant increase in both shooting frequency (0.58 shoots/explant) and regeneration efficiency (36.3%) was observed when compared to the empty vector control (0.013 shoots/explant, 1.3%). In addition, the introduction of r*GRF4–GIF1* into Valmaine significantly increased the shoot frequency (0.39 shoots/explant) and regeneration efficiency (26.8%) when compared to the control (0.02 shoots/explant, 2.4%). Therefore, grape *rGRF4–GIF1* increased the regeneration of all cultivars but the magnitude of this effect was greatest with the genotypes that regenerated poorly in its absence. The greatest enhancement was observed with regeneration efficiency of Salinas from 1.3 to 36%.

### Co-transformation strategies to generate transgenics with genes of interest using rGRF–GIF

We then tested the ability of a GRF–GIF fusion to increase the recovery rate of transgenic plants transformed with an independent T-DNA. To this end a construct containing the best performing GRF–GIF fusion (grape rGRF4–GIF1) and a dsRED reporter construct (*pLsUBI–dsRED–tLsUBI*) presented in separate strains of *A. tumefaciens,* were co-transformed into Cobham Green and Armenian 999 cultivars.

We also examined different antibiotic-selection treatments after co-transformation to identify a strategy for increasing the generation of transgenics with a gene of interest. Each genotype was co-transformed with the grape *rGRF4–GIF1* (BASTA resistance) and *pLsUBI–dsRED–tLsUBI* (kanamycin resistance), followed by selection on kanamycin (coTF Kan), BASTA (coTF BASTA), or kanamycin and BASTA (coTF Kan + BASTA) (Table 2). As a control, a fourth co-transformation of both genotypes was performed using an empty vector harboring BASTA resistance (pTB005) and *pLsUBI–dsRED–tLsUBI*, which was selected for transformants on kanamycin (coTF Control).

**Fig. 4:**
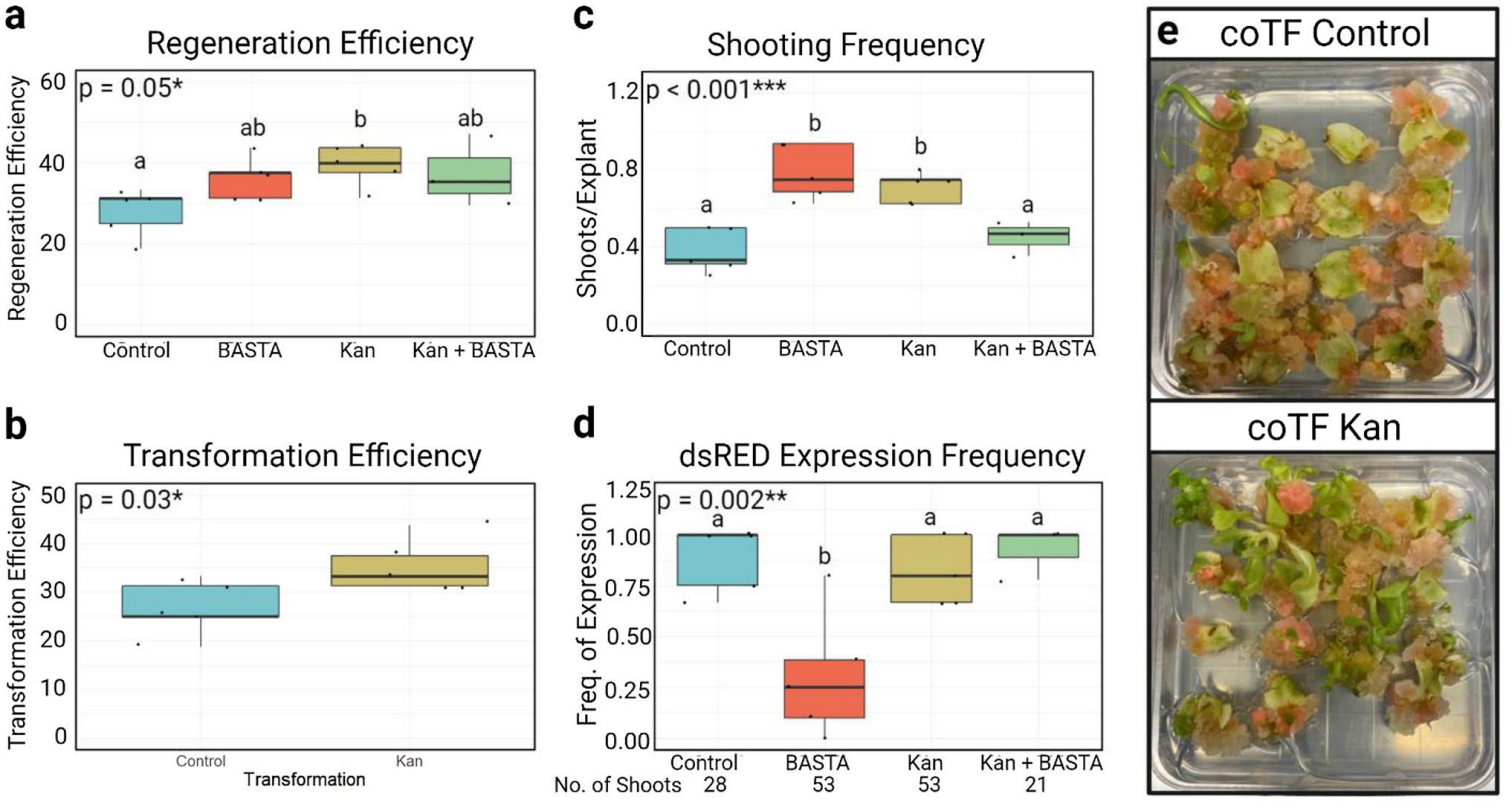
Summary of the co-transformation experiment in Cobham Green using *pLsUBI:dsRED:tLsUBI* + Grape r*GRF4–GIF1* selected on either kanamycin (Kan), BASTA^™^ (BASTA) or kanamycin + BASTA^™^ (Kan + BASTA) and *pLsUBI:dsRED:tLSUBI* + empty vector control (pTB005) selected on kanamycin (control). **a–d** Box plots representing regeneration efficiency (**a**), transformation efficiency (**b**), shooting frequency (**c**), and *dsRED* expression frequency (**d**) of each co-transformation. Letters above each boxplot represent pairwise significance differences (TukeyHSD, *α* = 0.05) and *p* values were calculated using a one-way ANOVA (*α* = 0.05) (**a**, **c**, **d**) or Welch’s *t* test (**b**). **e** Differences of regeneration in tissue culture between control (top) and Kan (bottom) co-transformations after 30 days in culture (color figure online)

**Fig. 5:**
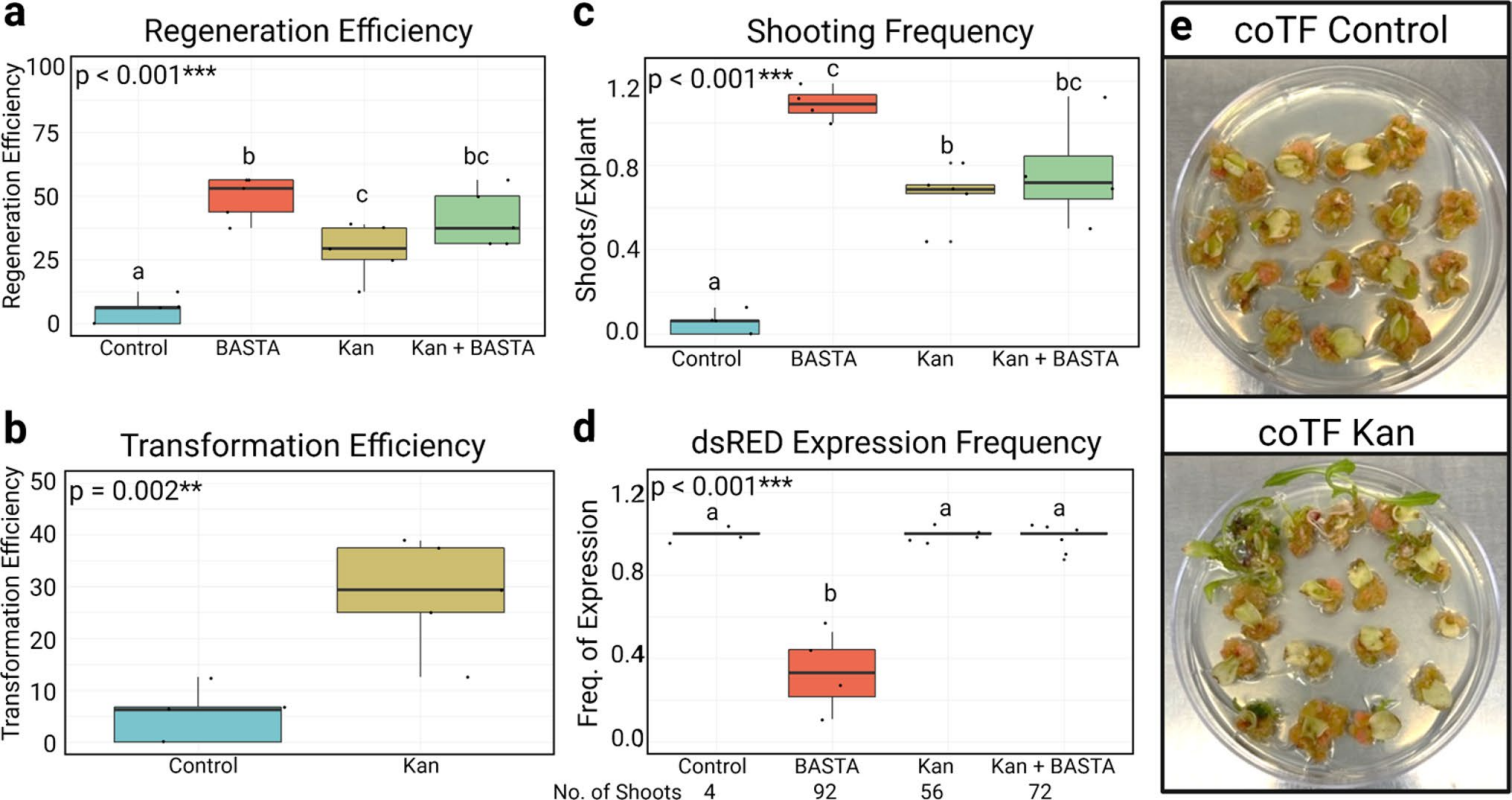
Summary of the co-transformation experiment in Armenian 999 using *pLsUBI:dsRED:tLsUBI* + Grape r*GRF4–GIF1* selected on either kanamycin (Kan), BASTA^™^ (BASTA) or kanamycin + BASTA^™^ (Kan + BASTA) and *pLsUBI:dsRED:tLSUBI* + empty vector control (pTB005) selected on kanamycin (control). **a**–**d** Box plots representing regeneration efficiency (**a**), transformation efficiency (**b**), shooting frequency (**c**), and *dsRED* expression frequency (**d**) of each co-transformation. Numbers below the x-axis (**d**) indicated the total number of shoot regenerated and phenotyped for dsRED expression over all the replications. Letters above each boxplot represent pairwise significance differences (TukeyHSD, *α* = 0.05) and *p* values were calculated using a one-way ANOVA (*α* = 0.05) (**a**, **c**, **d**) or Welch’s *t* test (**b**). **e** Differences of regeneration in tissue culture between control (top) and Kan (bottom) co-transformations after 35 days in culture

Co-transformation with *GRF–GIF* boosted regeneration efficiency and shooting frequency (Figs. 4 and 5, Supplemental Fig. 3) in both Cobham Green and Armenian 999. When compared to the coTF Control, the coTF Kan treatment resulted in a significant increase in regeneration efficiency by 8.4 and 23.5% in Cobham Green and Armenian 999, respectively (Figs. 4a and 5a). Since kanamycin selects for the T-DNA having dsRed, the increased regeneration translated directly into increased transformation frequency (Figs. 4b and 5b). As multiple shoots were regenerated from both cut sites on either side of individual explants, we then calculated the shooting frequency of each co-transformation (Figs. 4c and 5c). Shooting frequencies differed significantly between treatments for both genotypes. The coTF BASTA treatment resulted in the highest number of regenerated shoots in both Cobham Green and Armenian 999. In addition, the coTF Kan treatment had significantly increased frequencies of shooting in both genotypes, which were approximately twofold and 13-fold higher than the control coTF for Cobham Green and Armenian 999, respectively. In Armenian 999, the coTF Kan + BASTA treatment had a significantly higher regeneration efficiency and shooting frequency when compared to the coTF Control; however, no significant difference was detected between these treatments in Cobham Green.

We then checked for dsRED signal in different independent events from the four treatments (Figs. 4d and 5d). Almost 100% of shoots from coTF Control, CoTF Kan, and CoTF Kan + BASTA treatments had dsRED signal, consistent with the Kan resistant phenotype. However, the coTF Kan treatment produced a total number of shoots that was approximately twofold and 14-fold larger than the coTF control in Cobham Green and Armenian 999, respectively. On the other hand, only a small fraction of shoots selected on BASTA (coTF BASTA) showed dsRED signal. This treatment showed the largest shooting frequency (Figs. 4c and 5c), but likely that was due to the selection of *rGRF–GIF* events only.

Therefore, co-cultivation with two strains carrying *rGRF–GIF* or the gene of interest (*pLsUBI–dsRED-tLsUBI*) and selection only for the T-DNA with the gene of interest using kanamycin, led to a significant increase in both shooting frequency and regeneration efficiency in both genotypes. As observed previously, the increase in regeneration was greater in the genotype that did not readily regenerate in the absence of the GRF–GIF fusion.

**Fig. 6:**
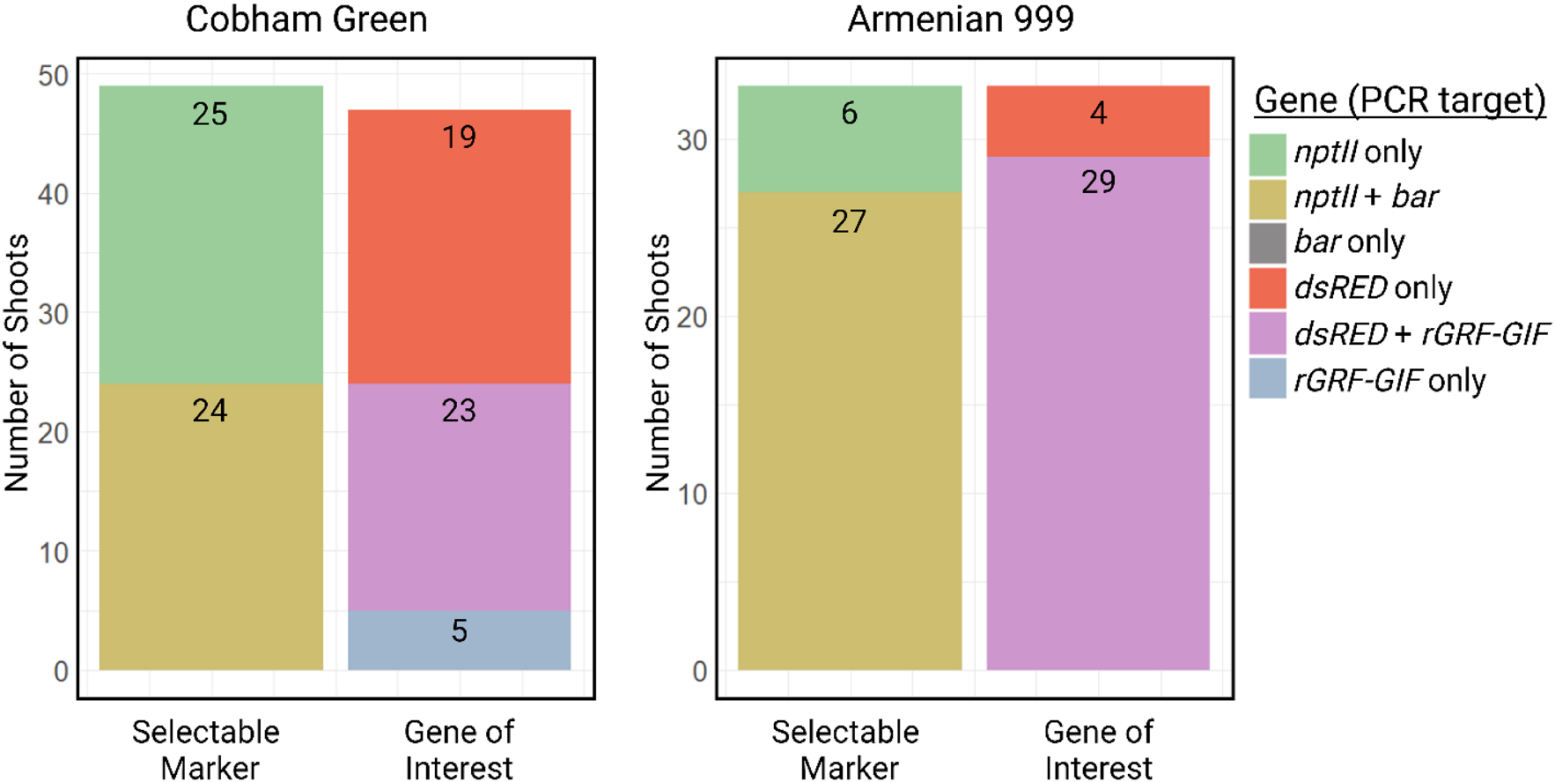
Frequency of regenerated Cobham Green and Armenian 999 shoots from the coTF Kan treatment containing each transgene after co-transformation with the grape r*GRF–GIF* and a gene of interest reporter, *pLsUBI–dsRED–tLsUBI*. The number of Cobham Green (left) or Armenian 999 (right) shoots that were PCR positive for selectable marker (kanamycin [*nptII*] and/or BASTA [*bar*]) and transgene (*dsRED* and/or *rGRF–GIF*). Each color represents the proportion of shoots that showed amplification of each specific gene target/s. The black numbers refer to the number of shoots PCR positive for each condition (color figure online)

To determine the proportion of transformants with only the gene of interest versus transformants with both transgenes, we checked for the presence of both T-DNAs by PCR of regenerants of both genotypes in the coTF Kan treatment (Fig. 6). A total of 49 Cobham Green and 33 Armenian 999 shoots from the coTF Kan treatment were screened by PCR. In Cobham Green, 24 (49%) showed amplification for the *GRF–GIF* transgene, resulting in a co-transformation efficiency of approximately 24% of explants co-cultivated (Fig. 6). This is similar to the dsRED expression-based estimate of co-transformation efficiency of the coTF BASTA treatment. In Armenian 999, 29 (88%) showed amplification for the *GRF–GIF* transgene resulting in co-transformation efficiency of 28%. The greater proportion of shoots containing the *GRF–GIF* with Armenian 999 compared to Cobham Green reflects the different amounts of enhancement of regeneration by GRF–GIF of the two genotypes. Therefore, co-transformation of an r*GRF–GIF* and a gene of interest in separate strains of *A. tumefaciens* increases the recovery of transgenic shoots harboring a gene of interest in multiple lettuce genotypes.

## Discussion

In vitro plant regeneration has been studied for decades; however, it remains a rate-limiting factor for *Agrobacterium*-mediated transformation for the development of transgenic plants and genome editing. Although considerable progress has been made in improving in vitro regeneration, efficient transformation is still limited in some crop species, including sunflower, cotton, and pepper (Gammoudi et al. 2018; Wu et al. 2004). In addition, regeneration and transformation efficiencies in many species, including *Lactuca* spp., are genotype dependent. Therefore, we tested *GRF–GIF* gene fusions from four species for their efficacy in increasing regeneration and transformation efficiency, and their ability to induce genotype-independent regeneration and transformation in lettuce.

Transformation using *GRF–GIF* gene fusions increased regeneration efficiency and shooting frequency in all tested lettuce genotypes. In both Cobham Green and Armenian 999, grape *rGRF4–GIF1* resulted in the highest frequencies of shoots and increased regeneration efficiency. The wild-type pepper and citrus *GRF4–GIF1* also increased shooting frequency in Cobham Green, but did not significantly increase regeneration efficiency. Furthermore, citrus *GRF4–GIF1* increased regeneration efficiency, but did not significantly increase shoot frequency in Armenian 999. Although both tomato and pepper are in the Solanaceae family and Euroasterids as lettuce, the wild-type tomato *GRF4#8–GIF1* did not increase shooting frequency or regeneration efficiency in any transformation experiment, while the pepper GRF–GIF did. Therefore, our limited data indicates that close taxonomic affinity between the source species for GRF and GIF genes and the target species may not be a prerequisite for efficacy of GRF–GIF fusions.

GRFs are post-transcriptionally regulated by miR396, and *GRF*s with mutated miR396 binding sites showed increased expression and activity in Arabidopsis (Debernardi et al. 2014, 2020; Rodriguez et al. 2010). Here, we observed that micro396 resistant *GRF–GIFs*, due to a mutated miR396 binding site, boosted regeneration when compared to the wild-type *GRF–GIF*. The grape *rGRF4–GIF1* exhibited the highest shooting frequencies and regeneration efficiencies of all fusions tested (Fig. 1). This is consistent with previous reports of the grape *rGRF4–GIF1* providing the largest increase in regeneration in citrus (Debernardi et al. 2020). Interestingly, introducing the same mutations into the miR396 binding site of tomato GRF–GIFs, rGRF4#8–GIF1 and rGRF4#12–GIF1, enhanced shooting frequencies and regeneration efficiencies compared to the wild-type tomato GRF4#8–GIF1 and the empty vector control. Therefore, in addition to *GRF* and *GIF* sequences, the regulation by miR396 seems to be an important determinant of GRF–GIF activity in lettuce. It would be interesting to perform transformations using resistant versions of the citrus and pepper *GRF*s to determine whether they boost regeneration efficiency and shooting frequency further compared to the wild-type versions and to levels higher than the tomato versions. Because the grape wild-type *GRF4–GIF1* was not available for this study, we cannot conclude if the observed increase in regeneration was due to the source species or solely the presence of a mutated binding site.

The grape *rGRF4–GIF1* increased regeneration in multiple diverse genotypes of lettuce that exhibit different tendencies to regenerate. This *GRF–GIF* increased regeneration efficiency and shooting frequency of four genotypes of lettuce, *L. serriola* acc. Armenian 999 (wild accession), and cultivars Cobham Green (butterhead), Salinas (crisphead), and Valmaine (romaine). The largest increase in regeneration was observed in genotypes that do not readily regenerate in the absence of *GRF–GIF*. This indicates that the GRF–GIF system could allow successful, genotype-independent transformations of diverse lettuce cultivars.

Transformation of the *L. sativa* cultivars with r*GRF–GIF* did not result in obvious changes in phenotype. However, transformation of Armenian 999 with the grape r*GRF4–GIF1* resulted in abnormal development and maturation of vegetative shoots and leaves (Supplemental Fig. 4). Armenian 999 is a wild accession with a different leaf morphology and is genetically more distant than the cultivars. It is possible that the abnormal phenotype is a result of an interaction between the genetic background and the inability of miR396 to post-transcriptionally regulate the transgene. However, this phenotype was not observed in Armenian 999 transformed with the two miRNA-resistant tomato fusions.

Finally, we developed a strategy to enhance the recovery of transgenic plants with a gene of interest through co-transformation with grape r*GRF4–GIF1*. The increased transformation efficiency may be a result from the accumulation of both the GRF–GIF induced regeneration from co-transformed cells in addition to the routinely recovered transgenics events seen with a single transformation. The increased transformation and regeneration efficiencies when *pLsUBI:dsRED:tLsUBI* was co-transformed with grape r*GRF4–GIF1* in separate *A. tumefaciens* strains and regenerated on media selecting for only the gene of interest could be readily applied to enhance the generation of transgenic plants using extant constructs without modification. When there had been integration of both T-DNAs, the *GRF–GIF* would have to be segregated away in the next generation, although this would be difficult if co-transformation of the two different T-DNAs resulted in co-integration at the same chromosomal position (Radchuk et al. 2005); this would not be feasible for clonally propagated crops.

The experiments in this paper extend previous reports and provide further evidence of the broad efficacy of the GRF–GIF system. The use of *GRF* and *GIF* genes derived from lettuce may boost regeneration efficiency even further. Fifteen *GRF* genes have been identified in lettuce, of which one has been shown to increase leaf size when ectopically expressed (Zhang et al. 2021). To our knowledge, no other studies have been conducted on identifying and characterizing *GIF* genes in lettuce. Identifying the closest lettuce homologs of the *GRF*s and *GIF*s used in this study, particularly the grape r*GRF4–GIF1*, may lead to insights about whether close taxonomic affinity increases the efficacy of GRF–GIF fusions to enhance regeneration rates. In the future, these studies could be extended to increase regeneration of other recalcitrant genotypes of lettuce, as well as to other recalcitrant crops of the Compositae family, such as sunflower.

## Supplementary Information

Below is the link to the electronic supplementary material.

Supplementary file1 (PDF 639 KB)

## Data Availability

Plasmids used in this study are available upon request.
