# Supplementary material for: GRF–GIF chimeric proteins enhance in vitro regeneration and Agrobacterium-mediated transformation efficiencies of lettuce (Lactuca spp.)
Source: Plant Cell Rep. 2023 Jan 25;42(3):629–43. doi: 10.1007/s00299-023-02980-4 (PMC10042933; doi:10.1007/s00299-023-02980-4)
Supplement: Supplementary file 1 — Supplementary file1 (PDF 639 KB) [file 299_2023_2980_MOESM1_ESM.pdf]

Supplemental Figures and Tables

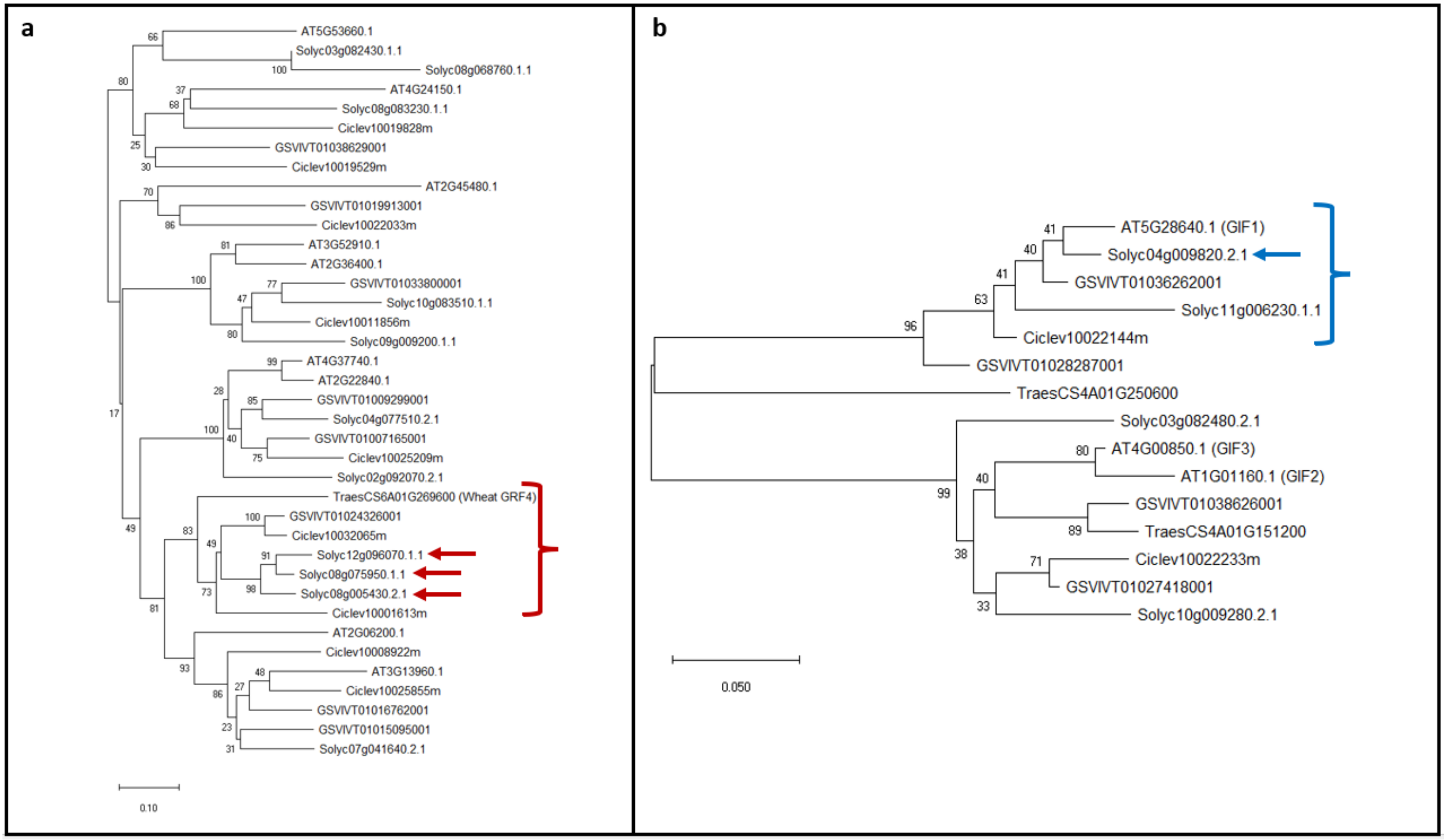

Supplemental Figure 1. Evolutionary relationships of *GRF* and *GIF* genes in analyzed taxa for identification of tomato *GRF* and *GIF* genes. **a**) The phylogenetic tree of GRF genes. The red bracket and arrows represents the tomato *GRF* genes, *GRF8* and *GRF12*, most closely related to the wheat and grape *GRF4* genes previously used. **b**) The phylogenetic tree of *GIF* genes used to identify the the closest tomato relative of the *Arabidopsis GIF1*. The blue bracket and arrow represents the tomato *GIF* gene, *GIF4*, selected for fusion constructs, and its closest relatives.

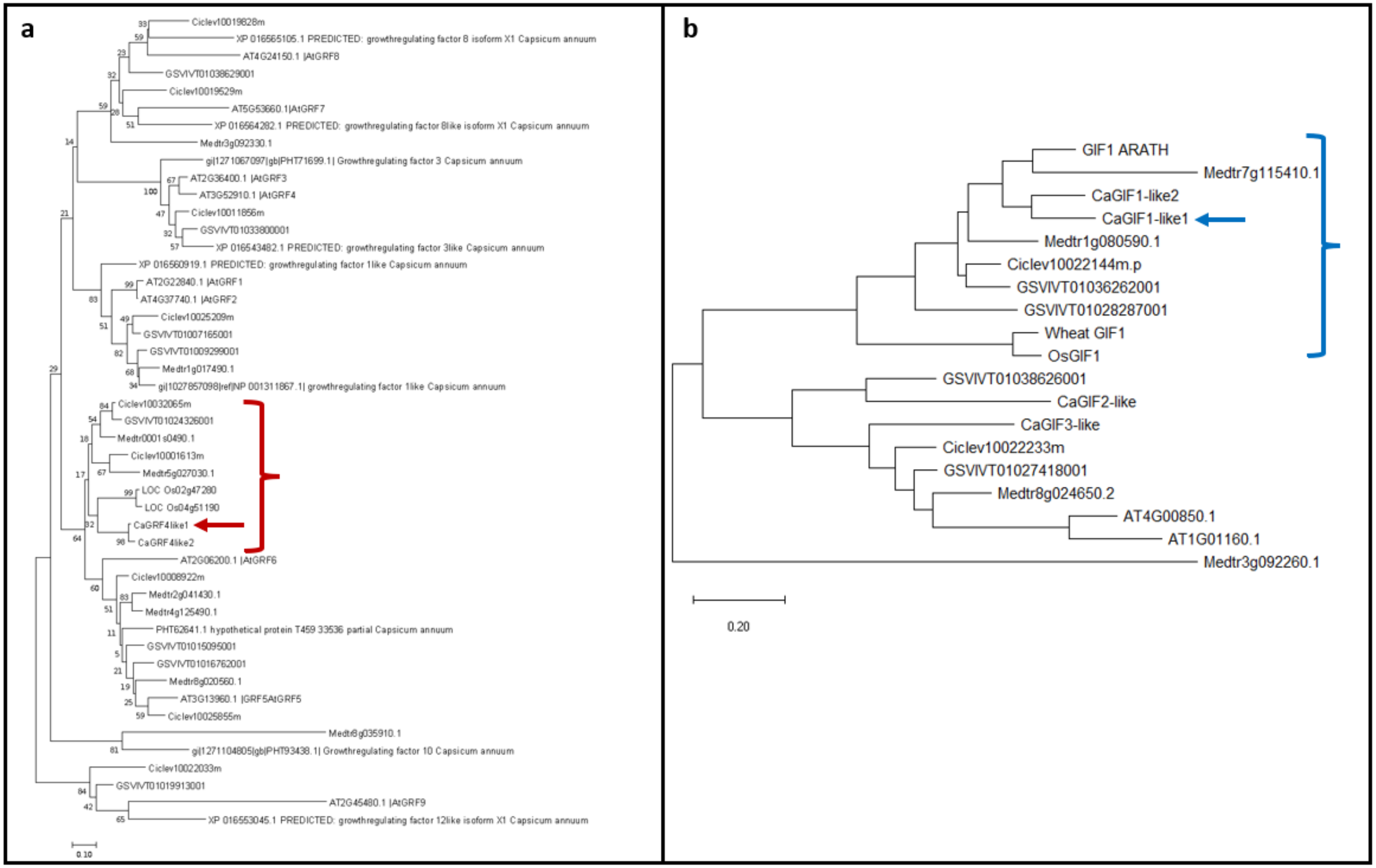

Supplemental Figure 2. Evolutionary relationships of *GRF* and *GIF* genes in analyzed taxa for identification of pepper *GRF* and *GIF* genes. **a**) The phylogenetic tree of *GRF* genes. The red bracket and arrow represents the pepper GRF gene most closely related to the grape *GRF4* previously used. **b**) The phylogenetic tree of *GIF* genes used to identify the the closest pepper relative of the *Arabidopsis GIF1*. The blue bracket and arrow represents the pepper *GIF* gene, *GIF1*-like1, selected for fusion constructs, and its closest relatives.

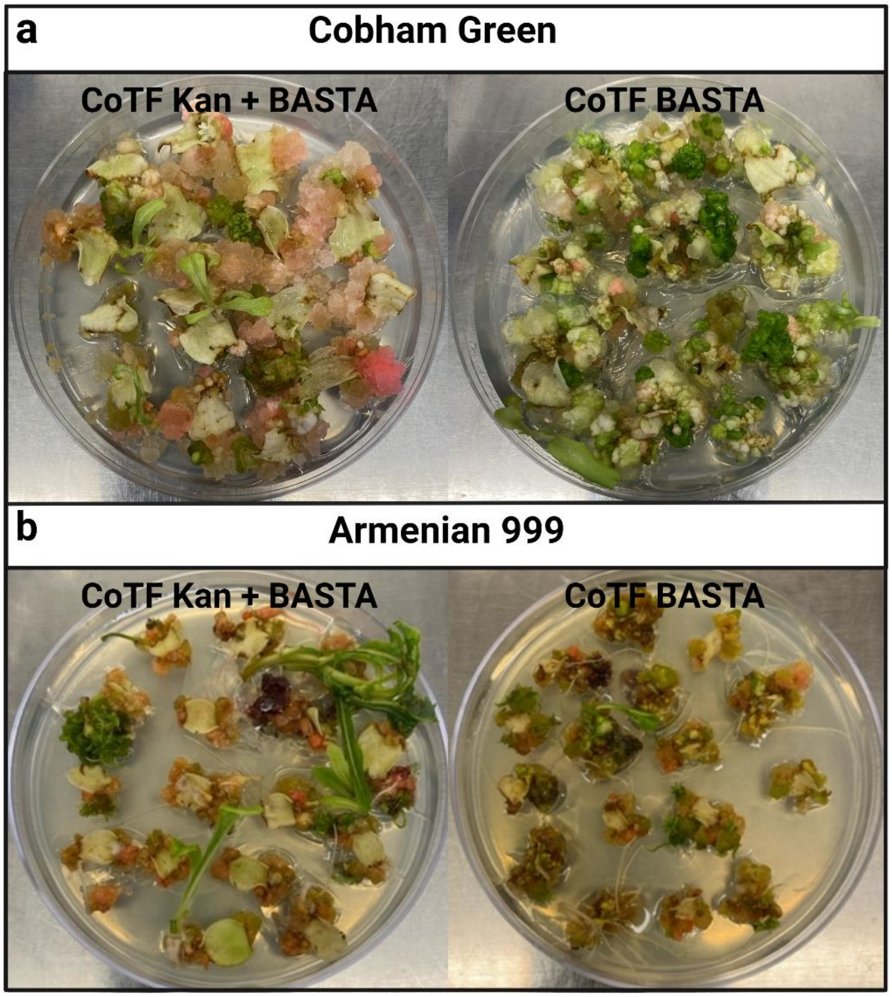

Supplemental Figure 3. Regeneration of Cobham Green and Armenian 999 after co-transformation with *pLsUBI:dsRED:tLsUBI* and Grape r*GRF4-GIF1*. a) Regeneration rates of Cobham Green selected on Kanamycin and BASTA (coTF Kan + BASTA) and BASTA only (CoTF BASTA). b) Regeneration rates of Armenian 999 selected on Kanamycin and BASTA (coTF Kan + BASTA) and BASTA only (CoTF BASTA).

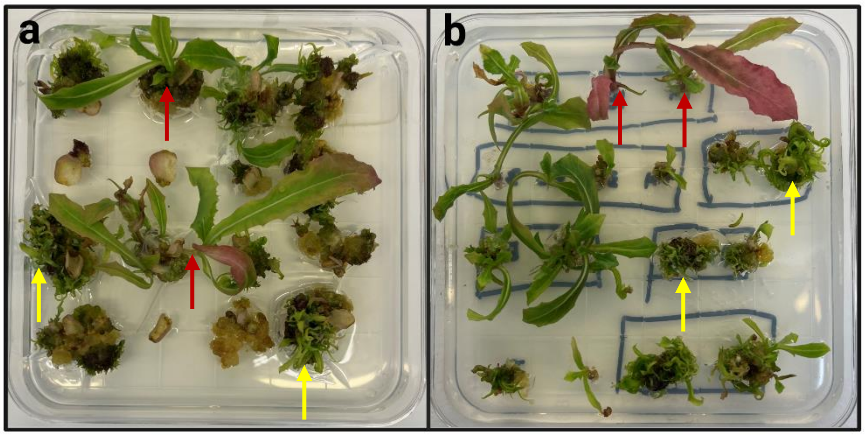

Supplemental Figure 4. Abnormal regeneration phenotype observed in Armenian 999 cultures after transformation with the grape r*GRF4-GIF1*. Red arrows represent normal shoot regeneration and yellow arrows represent abnormal shoot regeneration. a) Armenian 999 culture after 40 days on induction medium. b) Armenian 999 culture after 6 days on elongation medium (46 total days in culture).

Supplemental Table 1. Groups of transformations with *GRF-GIF* performed with construct name, construct components, and lettuce genotype. Blue highlighted transformations were performed to identify which GRF-GIF construct resulted in the highest increase in regeneration in lettuce. Orange highlighted boxes were performed to observe if miRNA396 resistant GRF-GIFs increased regeneration when compared to wildtype GRF-GIFs. The green highlighted transformations were performed to identify if the introduction of a GRF-GIF result in genotype independent regeneration in lettuce. Transformations were performed in Cobham Green (CG), Armenian 999, Valmaine, and Salinas.

Construct	Vector Backbone	Components	Genotype(s)	Selection
pTB005	pEG100	Empty Vector	CG, Armenian 999	BASTA
JD761	pEG100	Tomato *GRF4#8-GIF1*	CG, Armenian 999	BASTA
JD638	pEG100	Grape r*GRF4-GIF1*	CG, Armenian 999	BASTA
JD689	pEG100	Citrus *GRF-GIF*	CG, Armenian 999	BASTA
pTH1903	pEG100	Pepper *GRF4-GIF1*	CG, Armenian 999	BASTA
JD641	pGWB14	Empty Vector	CG, Armenian 999	Kan
JD746	pGWB14	Tomato *GRF4#8-GIF1*	CG, Armenian 999	Kan
JD747	pGWB14	Tomato r*GRF4#8-GIF1*	CG, Armenian 999	Kan
JD749	pGWB14	Tomato r*GRF4#12-GIF1*	CG, Armenian 999	Kan
pTB005	pEG100	Empty Vector	CG, Armenian 999, Valmaine, Salinas	BASTA
JD638	pEG100	Grape r*GRF4-GIF1*	CG, Armenian 999, Valmaine, Salinas	BASTA

Supplemental Table 2. Primer names, sequences, and PCR conditions used for amplification of the transgene for each transformation.

Primer Name	Primer Target	Primer sequence	Amplicon size (bp)	Annealing Temp. (°C)	Extension Time (sec)
TB166	Grape r*GRF4-GIF1* Fw	5’ ATGGACTCGCCTTTGGACAG3’	319	59.4	45
TB167	Grape r*GRF4-GIF1* Rv	5’GACCGAGAGCTGGTTGTTGA 3’			
TB68	*nptII* (JD641) Fw	5’ GGTGCCCTGAATGAACTCCA 3’	448	59.0	45
TB69	*nptII* (JD641) Rv	5’ AAAAGCGGCCATTTTCCACC 3’			
TB145	*Bar* (pTB005) Fw	5’ CAGTTCCCGTGCTTGAAGC 3’	307	58.9	45
TB146	*Bar* (pTB005) Rv	5’ CGCTATCCCTGGCTCGTC 3’			
TB109	*dsRED* Fw	5’ CCGACATCCCCGACTACAAG 3’	151	58.7	30
TB85	*dsRED* Rv	5’ ACGCCGATGAACTTCACCTT 3’			

Supplemental Table 3. Sequences of primers used for introduction of silent mutations in the miR396 binding site to develop resistant GRF-GIF fusions.

Primer Name	Primer Sequence
Fw_Gw	5’ GGGGACAAGTTTGTACAAAAAAGC 3’
Rev_Gw	5’ GGGGACCACTTTGTACAAGAAAGC 3’
rGRF#8Fw	5’ TCTAGAAAACCGGTCGAATCTCAATCTA 3’
rGRF#8Rev	5’ TCGACCGGTTTTCTAGAACGGTTGCGG 3’
rGRF#12Fw	5’ TCTAGAAAACACGTCGAATCTCAATCGA 3’
rGRF#12Rev	5’ TCGACGTGTTTTCTAGAACGGTTGCGG 3’
